# SARS-CoV-2 Delta Variant Decreases Nanobody Binding and ACE2 Blocking Effectivity

**DOI:** 10.1021/acs.jcim.1c01523

**Published:** 2022-05-09

**Authors:** Mert Golcuk, Aysima Hacisuleyman, Sema Zeynep Yilmaz, Elhan Taka, Ahmet Yildiz, Mert Gur

**Affiliations:** † Department of Mechanical Engineering, Istanbul Technical University (ITU), 34437 Istanbul, Turkey; ‡ Institute of Bioengineering, Swiss Federal Institute of Technology (EPFL), 1015 Lausanne, Switzerland; § Physics Department, University of California, Berkeley, California 94720, United States; ∥ Department of Molecular and Cellular Biology, University of California, Berkeley, California 94720, United States

## Abstract

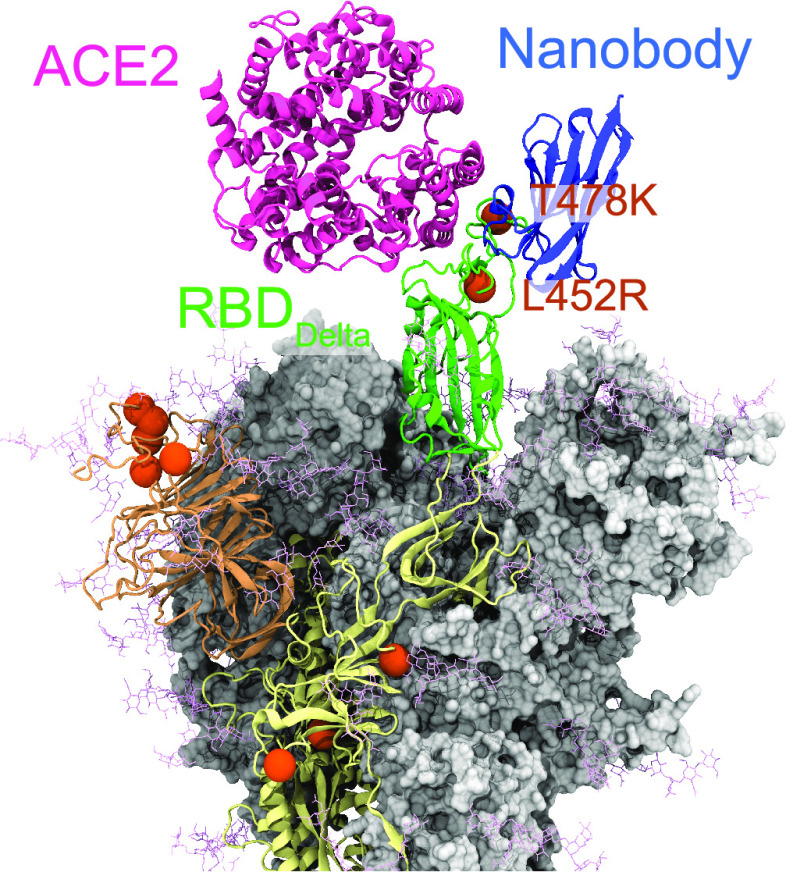

The Delta variant spreads more rapidly than previous variants of SARS-CoV-2. This variant comprises several mutations on the receptor-binding domain (RBD_Delta_) of its spike glycoprotein, which binds to the peptidase domain (PD) of angiotensin-converting enzyme 2 (ACE2) receptors in host cells. The RBD–PD interaction has been targeted by antibodies and nanobodies to prevent viral infection, but their effectiveness against the Delta variant remains unclear. Here, we investigated RBD_Delta_–PD interactions in the presence and absence of nanobodies H11-H4, H11-D4, and Ty1 by performing 21.8 μs of all-atom molecular dynamics simulations. Unbiased simulations revealed that Delta variant mutations strengthen RBD binding to ACE2 by increasing the hydrophobic interactions and salt bridge formation, but weaken interactions with H11-H4, H11-D4, and Ty1. Among these nanobodies H11-H4 and H11-D4 bind RBD without overlapping ACE2. They were unable to dislocate ACE2 from RBD_Delta_ when bound side by side with ACE2 on RBD. Steered molecular dynamics simulations at comparable loading rates to high-speed atomic force microscopy (AFM) experiments estimated lower rupture forces of the nanobodies from RBD_Delta_ compared to ACE2. Our results suggest that existing nanobodies are less effective to inhibit RBD_Delta_–PD interactions and a new generation of nanobodies is needed to neutralize the Delta variant.

## Introduction

Nanobodies are promising alternatives to conventional antibodies because they are smaller in size (15 kDa),^1^ have a similar affinity to conventional antibodies, enter the cell more readily, and can be mass-produced at a lower cost.^2,3^ The small size of the nanobodies also enables them to bind epitopes normally not accessible to conventional antibodies,^4^ including conserved viral domains often masked by glycan shields.^5^ Currently, there are more than 180 neutralizing nanobodies targeting the SARS-CoV-2 spike (S) glycoprotein^6^ and the structures of more than 30 nanobodies have been recently determined.^7^ Most of these nanobodies have shown promising neutralizing activity against wild-type (WT) SARS-CoV-2,^2,3,5,8−16^ but their effectivity against the Delta variant remains to be elucidated.

**Figure 1:**
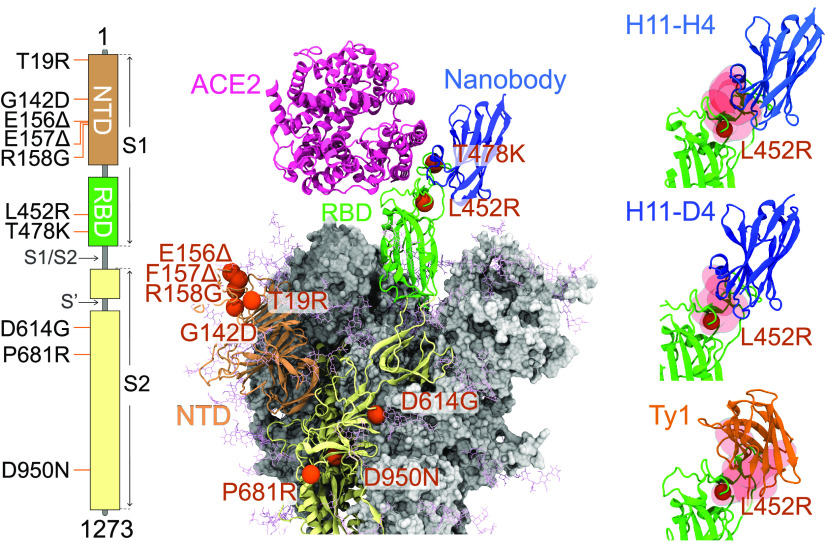
Locations of the mutations observed in the Delta variant are shown on the S protein structure. Sites of the 10 mutations of the Delta variant are highlighted with orange spheres. Crystal structures of nanobodies (PDB IDs: 6ZBP,^9^6YZ5,^9^ and 6ZXN(2)) and ACE2 (PDB ID: 6M0J(33)) are docked onto RBD_Delta_.

Delta (B.1.617.2) variant was the most dominant variant between April and December 2021 with the highest number of reported cases.^17−19^ The Delta variant comprises 10 mutations on the homotrimeric S protein (Figure 1), which is the critical protein that mediates host cell entry of the virus via binding of its receptor-binding domain (RBD) to the angiotensin-converting enzyme 2 (ACE2) receptor of the host cells. Two of these mutations are located on the ACE2 binding surface of RBD (L452R and T478K), while five mutations are located on the N terminal domain (NTD) surface (T19R, G142D, E156del, F157del, and R158G), and three mutations are located in S2 (D614G, P681R, and D950N).^17,20,21^ These mutations are positioned on the binding interfaces for a wide range of antibodies and nanobodies, potentially affecting their binding strengths. Consistent with this view, recent experimental studies revealed a substantial decrease in the neutralization activity of many neutralizing antibodies against the Delta variant, including those formed by major vaccines, such as mRNA vaccines (mRNA-1273 and BNT162b2)^22,23^ and adenovirus vector vaccines (Sputnik V and ChAdOx1).^24,25^ Only a few antibodies retained their neutralization activity.^20,26−28^ The molecular mechanism underlying loss in antibody and nanobody effectivity have been investigated for various SARS-CoV-2 variants, including Alpha and Beta.^29−31^ However, the underlying mechanism of the reduced effectiveness of the nanobodies against the Delta variant is not well understood.

In our recent study, we performed extensive molecular dynamics (MD) simulations of the nanobodies H11-H4, H11-D4, and Ty1 in complex with WT, Alpha, and Beta variants of the RBD of SARS-CoV-2 S protein.^29^ We showed that the Delta variant mutation L452R is located at the hydrophobic core of the nanobody–RBD interface for WT SARS-CoV-2. Thus, it remains to be explored how this mutation affects the binding strength. A recent *in vitro* and *in silico* study^32^ estimated that the S protein of the Delta variant of SARS-CoV-2 binds to ACE2 with similar strength compared to WT and weaker than the Alpha variant. Considering that Delta became more dominant than the Alpha variant, it is also critical to investigate how these novel mutations affect the effectiveness of antibodies and nanobodies to prevent S–ACE2 interactions.

To explore the effect of Delta variant mutations on neutralizing nanobodies, we performed all-atom MD simulations of the RBD of S protein of the Delta variant (RBD_Delta_) in complex with either the peptidase domain (PD) of human ACE2 or nanobodies H11-H4, H11-D4, and Ty1. ACE2-bound H11-H4 and H11-D4 do not sterically overlap with the RBD–ACE2 binding site, while Ty1 sterically overlaps with ACE2. Thus, we also simulated RBD_Delta_ in complex with both ACE2 and either H11-H4 or H11-D4. These nanobodies were not able to dislocate ACE2 from RBD_Delta_ upon binding, indicating lower neutralizing activity against this variant. In addition, we simulated the detachment of the nanobodies from RBD at loading rates directly comparable to high-speed atomic force microscopy (AFM) studies.^34^ Our simulations totaling 21.8 μs in length show that the Delta variant mutations increase the binding strength of RBD_Delta_ to ACE2 while reducing the binding strength of the nanobodies to RBD_Delta_.

## Methods

### System Preparations for MD Simulations

The structure of SARS-CoV-2 S protein RBD bound with ACE2 at 2.45 Å resolution (PDB ID: 6M0J(33)) was used as a template for the MD simulations of the RBD–ACE2 complex. To obtain the Delta variant structure, L452R and T478K mutations were performed on the RBD using the Mutator plugin of Visual Molecular Dynamics (VMD).^35^ Crystal structures of nanobodies (PDB IDs: 6ZBP,^9^6YZ5,^9^ and 6ZXN(2)) were used for constructing the solvated H11-H4-RBD, H11-D4-RBD, and Ty1-RBD systems, respectively. The protonation states of the titratable residues were predicted using the PROPKA web server.^36,37^ Titratable residues were left in their dominant protonation state at pH 7.0. The chloride ion, zinc ion, glycans, and water molecules present in the structures were kept. Full-length glycans are not visible in the crystal structure. Thus, glycan models^38^ were added to the structures. For conventional MD (cMD) simulations, each system was solvated in a water box (using the TIP3P water model) with a 25 Å cushion in each direction (50 Å water cushion between the protein complexes and their periodic images). For steered MD^39^ (SMD) simulations, systems were solvated having a 50 Å cushion along the pulling direction to create enough space for unbinding simulations and a 15 Å cushion in all other directions. Ions were added to neutralize the system and NaCl concentration was set to 150 mM. The size of solvated systems was ∼150,000, ∼120,000, and ∼280,000 atoms for cMD, SMD, and RBD_Delta_–PD–nanobody simulations, respectively. All system preparation steps were performed in VMD.

### MD Simulation Details

All MD simulations were performed under N, P, and T conditions in NAMD 2.14^40^ using the CHARMM36^41^ force field with a time step of 2 fs. Using the Langevin Nosé–Hoover method with an oscillation period of 100 fs and a damping time scale of 50 fs, the pressure was maintained at 1 atm. Using Langevin dynamics with a damping coefficient of 1 ps^–1^, the temperature was kept at 310 K. For van der Waals interactions, a 12 Å cutoff distance was used. To calculate long-range electrostatic interactions, the particle-mesh Ewald method was used. In all simulations, periodic boundary conditions were applied. First, each system was minimized for 10,000 steps and subsequently equilibrated for 2 ns by keeping the protein fixed. A second minimization–equilibration cycle was performed: the complete system was minimized for additional 10,000 steps without fixing the protein, followed by 4 ns of equilibration by applying harmonic constraints on C_α_ atoms. As a final step before production runs, the constraints were released and the system was equilibrated for additional 4 ns, during which the root-mean-square deviation (RMSD) values converged. These simulations are expected to account for the structural differences due to the radically different thermodynamic conditions of crystallization solutions and MD simulations.^42^ After the final equilibration step, each simulation (Table S1) was run for 400 ns length to determine interactions between proteins. MD simulations were performed in Longhorn, Expanse, and Stampede2 using a total of ∼9 million core-hours.

### Criteria for Interaction Analysis

Using MD simulation trajectories, we determined salt bridges, hydrogen bonds, and electrostatic and hydrophobic interactions between the RBD_Delta_ and PD of human ACE2. For salt bridge formation, a cutoff distance of 6 Å between the basic nitrogen and acidic oxygen was used.^43,44^ For hydrogen bond formation, a cutoff distance of 3.5 Å between hydrogen bond donor and acceptor and a 30° angle between the hydrogen atom, the donor heavy atom, and the acceptor heavy atom were used.^44,45^ Interaction pairs that did not satisfy the angle criterion but satisfied the distance criterion was classified as electrostatic interactions. For hydrophobic interactions, a cutoff distance of 8 Å between the side-chain carbon atoms was used.^46−48^ Observation frequencies were classified as high and moderate for interactions that occur in 49% and above and between 15 and 48% of the total trajectory, respectively.^29,49^ All reported changes in interactions frequency classifications due to the Delta mutations were statistically different from that of the RBD of WT SARS-CoV-2 S protein (RBD_WT_ ) (Student’s *t*-test, *p* < 0.05).^29,49^ Pairwise interactions with observation frequencies below 15% were excluded from further analysis.

### SMD Simulations

Steered and fixed atoms were selected as the C_α_ atoms at the nanobody–RBD and RBD–ACE2 interface (Table S2). The vector pointing from the center of mass of fixed atoms to the center of mass of steered atoms was selected as a pulling direction (Figure S1). Each SMD simulation was performed until the rupture event is observed for the nanobody. Four different starting conformations (from 140, 160, 180, and 200 ns) were taken from each cMD simulation to perform SMD.

## Results and Discussion

### Interactions of Delta Variant RBD with ACE2

**Figure 2:**
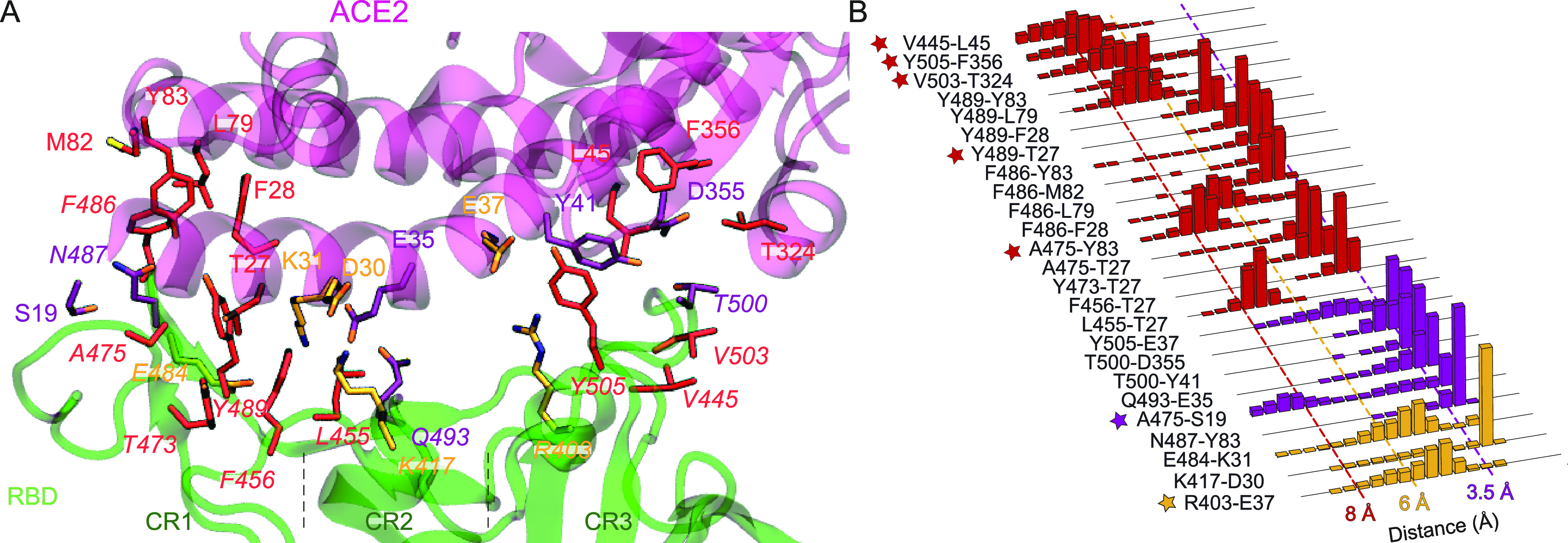
Interactions between SARS-CoV-2 S protein RBD and ACE2 PD. (A) Salt bridges, hydrogen bonds, and hydrophobic interactions between RBD_Delta_ and PD are shown in orange, purple, and red, respectively. RBD residue indices are shown in italic. Electrostatic interactions are listed in Table S3. (B) Normalized distributions of the distances between the amino-acid pairs that form salt bridges (orange), hydrogen bonds (purple), and hydrophobic interactions (red). The interactions newly observed for RBD_Delta_ with ACE2 are marked with stars.

In our previous study, we determined the interaction network between RBD–PD by performing MD simulations of the RBD_WT_ in complex with the PD of human ACE2.^49^ To determine how RBD_Delta_ interacts with PD, we performed MD simulations of the RBD_Delta_–PD complex. L452R and T478K mutations were manually introduced to the RBD_WT_ structure (PDB ID: 6M0J)^33^ to obtain the RBD_Delta_ structure. Two sets of cMD simulations, each of 400 ns in length (Table S1), were performed to determine the salt bridges, hydrogen bonds, and electrostatic and hydrophobic interactions (see Methods), and the results were compared to that of RBD_WT_ (Figures 2 and S2, and Table S3). Two sets of cMD simulations were combined and observation frequencies were reported based on this 800 ns long trajectory. We detected one new salt bridge (R403-E37), four new hydrophobic (A475-Y83, Y489-T27, V503-T324, and Y505-F356), and two new electrostatic interactions at high frequencies in the RBD_Delta_–PD complex compared to RBD_WT_ (Table 1). However, two high-frequency hydrogen bond interactions (Q493-E35 and T500-D355) in RBD_WT_–PD were observed in moderate frequencies in the RBD_Delta_–PD complex (Table 1). In addition, we detected one new hydrogen bond (A475-S19), one new hydrophobic (V445-L45), and three new electrostatic interactions at moderate frequencies in the RBD_Delta_–PD complex (Table S3). Three hydrogen bond interactions observed with moderate frequencies for RBD_WT_ (Y449-D38, Q498-Q42, and Q498-K353) were either observed at low frequencies or completely disappeared in RBD_Delta_. For each system, observation frequencies of hydrogen bonds and electrostatic interactions differed moderately between two sets of simulations, but similar frequencies were detected for most hydrophobic interaction and salt bridge formations (Table S4).

**Table 1:** Net Changes in the Number of Detected High-Frequency Interactions between RBD and PD due to Delta Variant Mutations^a^

	**salt bridges**	**hydrogen bonds**	**hydrophobic interactions**	**electrostatic interactions**
ACE2	+1 (3/2)	–2 (1/3)	+4 (15/11)	+2 (3/1)
H4	0 (1/1)	–2 (3/5)	–1 (9/10)	0 (1/1)
D4	0 (1/1)	–4 (1/5)	–2 (4/6)	0 (0/0)
Ty1	0 (0/0)	–4 (1/5)	–3 (15/18)	0 (0/0)

a Parentheses show (number of interactions with RBD_Delta_/number of interactions with RBD_WT_).

For RBD_WT_, we divided the RBD–ACE2 interaction surface into three contact regions (CR1–3) and proposed that the RBD–ACE2 interaction is primarily stabilized by hydrophobic interactions in CR1.^49,50^ Due to the Delta variant mutations, CR1 gains two additional hydrophobic interactions (A475-Y83 and Y489-T27), while CR2 remains unaffected. Remarkably, CR3 gains one salt bridge (R403-E37) and two hydrophobic interactions (V503-T324 and Y505-F356) while losing three hydrogen bonds (Y449-D38, Q498-Q42, and Q498-K343) with PD. In our previous study,^49^ we highlighted the role of CR1 in anchoring ACE2 and the importance of blocking its surface for S protein inhibition. Because CR3 also forms an extensive interaction network in the Delta variant, it may also be critical to target CR3 to prevent S–ACE2 interactions in the Delta variant.

### Interactions of RBD_Delta_ with H11-H4, H11-D4, and Ty1 Nanobodies

**Figure 3:**
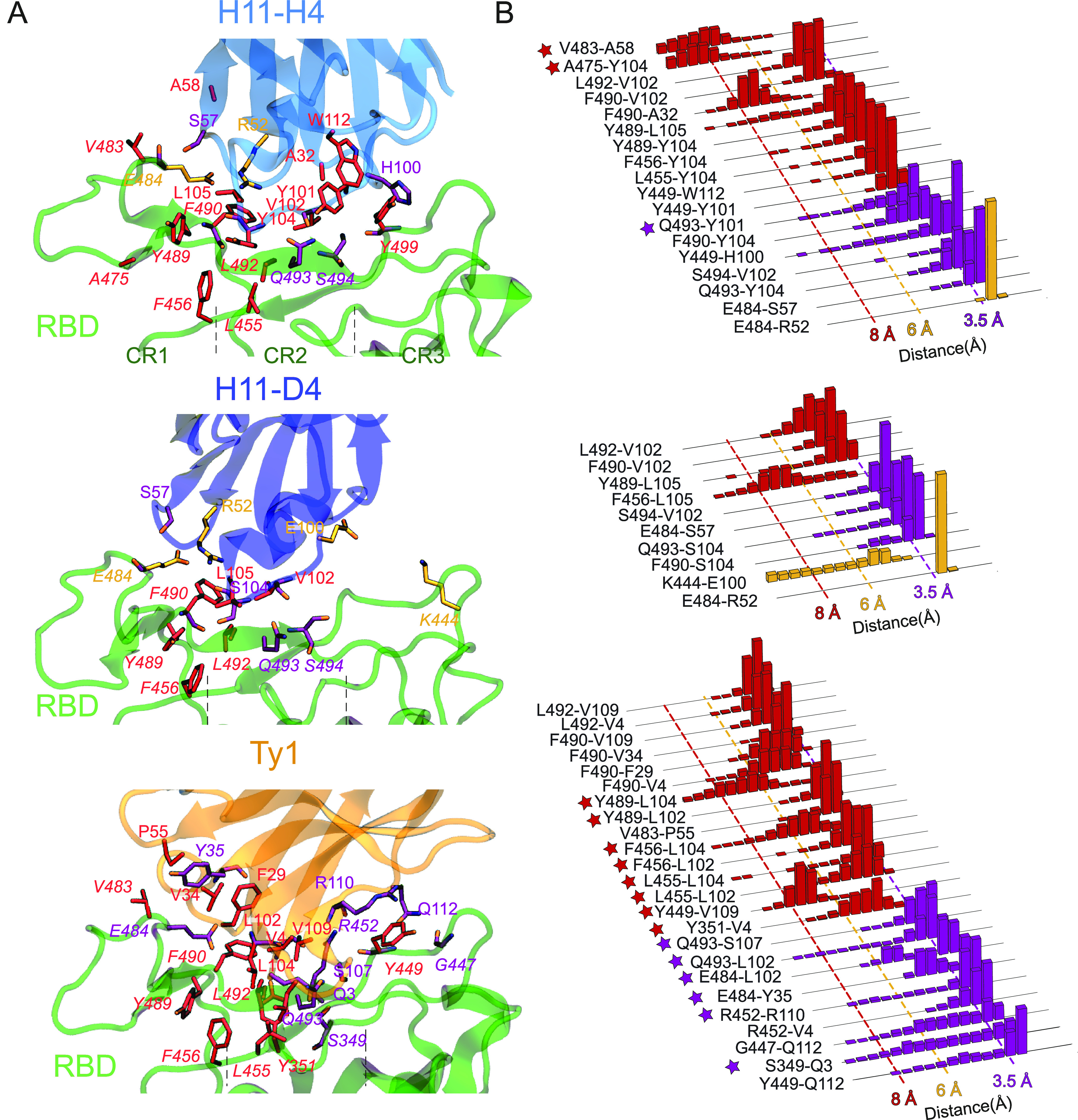
Interactions between SARS-CoV-2 S protein RBD and nanobodies. (A) Salt bridges (orange), hydrogen bonds (purple), and hydrophobic interactions (red) between RBD_Delta_ and nanobodies are shown. RBD residue indices are shown in italic. Electrostatic interactions are listed in Table S3. (B) Normalized distributions of the distances between the amino-acid pairs that form salt bridges, hydrogen bonds, and hydrophobic interactions are shown in orange, purple, and red, respectively. The interactions newly observed for RBD_Delta_ with nanobodies are marked with stars.

To investigate the interactions of the RBD_Delta_ with nanobodies, we introduced Delta variant mutations to the co-structures of RBD in complex with H11-H4,^9^ H11-D4,^9^ and Ty1.^2^ For each RBD_Delta_–nanobody complex, two sets of cMD simulations, each of 400 ns length (Table S1), were performed to determine pairwise interactions. Although H11-H4 and H11-D4 stayed bound to RBD_Delta_ in a binding mode throughout the simulations, Ty1 was observed to leave its original binding mode in one of the simulations (∼100 ns into the simulation) and sampled various binding modes within 400 ns (Movie S1 and Figure S3). Thus, the interaction network for Ty1 is reported based on a single trajectory where it kept its original binding mode, while both trajectories are used for H11-H4 and H11-D4 (Figures 3 and S2 and Table S3).

A comparison of H11-H4’s high-frequency interactions with RBD_WT_ and RBD_Delta_ shows that two hydrogen bonds (Y449-H100 and F490-Y104) and one hydrophobic interaction (L452-V102) disappeared upon Delta mutations (Table 1). While L452-V102 disappeared completely, Y449-H100 and F490-Y104 were observed only with moderate frequencies for the Delta variant (Table S3). In addition, we detected one new hydrogen bond (Q493-Y101), two new hydrophobic (A475-Y104 and V483-A58) and two new electrostatic interactions at moderate frequencies (Table S3).

Similarly, for H11-D4, three of its hydrogen bonds (N450-E100, E484-S57, and S494-V102) and two of its hydrophobic interactions (L452-V102 and L455-L105) either completely disappeared (N450-E100, L452-V102, and L455-L105) or were observed at a lower frequency (E484-S57 and S494-V102) with RBD_Delta_ (Table 1 and Figure S2). One high-frequency hydrogen bond (Q493-S104) was observed in moderate frequency. However, we detected six new electrostatic interactions at moderate frequencies (Table 1), while two electrostatic interactions were only observed at low frequencies.

For Ty1 nanobody, based on the single trajectory where binding to RBD_Delta_ was observed, two of its hydrogen bonds (V483-V34 and S494-S107) and 11 of its hydrophobic interactions (L452-V102, I472-F29, I472-P55, I472-V34, L452-V4, L452-V109, L492-I100, L492-F29, F490-I100, F490-L6, and F490-L104) either completely disappeared or were observed at low frequency (Table 1). We detected eight new hydrophobic interactions (Y351-V4, Y449-V109, L455-L102, L455-L104, F456-L102, F456-L104, Y489-L102, and Y489-L104) at high frequencies (Figure 3 and Table 1). Thus, the total number of hydrogen bonds and hydrophobic interactions observed at high frequency decreased by 4 and 3, respectively, while the total number of high-frequency electrostatic interactions increased by 1 for the Delta variant (Table 1). In addition, we detected six new hydrogen bonds (S349-Q3, R452-R110, E484-Y35, E484-L102, Q493-L102, and Q493-S107), one new hydrophobic (Y449-V109) and six new electrostatic interactions at moderate frequencies (Table S3). One hydrogen bond (E484-N56) and six electrostatic interactions observed with moderate frequencies completely disappeared. For the second set of Ty1-RBD_Delta_ MD simulation, a stable binding was not observed, and all interactions observed between Ty1 and RBD_WT_ completely disappeared.

### H11-H4 and H11-D4 Are Not Able to Abrogate ACE2 Binding to RBD_Delta_

**Figure 4:**
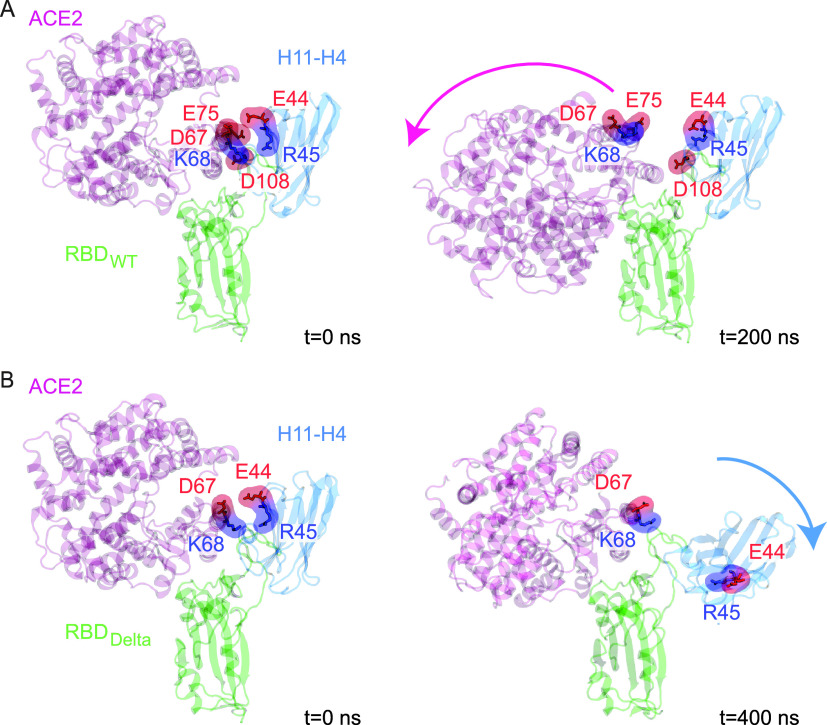
Effect of Delta variant RBD mutations on the H11-H4’s ability to dislocate ACE2. Electrostatic repulsion between ACE2 and H11-H4 upon H11-H4 docking on (A) RBD_WT_^29^ and (B) RBD_Delta_. Neighboring ACE2 and H11-H4 residues with identical charges are highlighted in surface representation in red (negatively charged) and blue (positively charged). H11-H4 binding resulted in a 95% decrease in pairwise interaction observed between ACE2–RBD_WT_,^29,49^ while H11-H4 lost 54% of its interaction when bound to RBD_Delta_ with ACE2 side by side.

To investigate if H11-H4 or H11-D4 binding can disrupt RBD_Delta_–PD interactions, we performed three sets of 400 ns cMD simulations of each RBD_Delta_–PD–nanobody complex (Table S1). Nanobodies were manually docked onto the complex (PDB ID: 6M0J(33)) using the RBD–nanobody structure coordinates (PDB IDs: 6ZBP(9) and 6YZ5(9)). Although we previously showed that these nanobodies can dislocate PD from RBD_WT_ via the repulsion of identically charged residues,^29^ they were unable to dislocate PD from RBD_Delta_ in any of our simulations (Figures 4 and S4). In addition, H11-H4 was dislocated from its RBD binding pose in all three simulations (Movie S2) due to the repulsion of identically charged residues of ACE2 and H11-H4 (ACE2 D67 with H11-H4 E44 and ACE2 K68 with H11-H4 R45, Figure 4) when ACE2 and H11-H4 are bound to RBD side by side. Therefore, while ACE2 loses the electrostatic tug-of-war against H11-H4 to bind RBD_WT_,^29^ it outcompetes H11-H4 on RBD_Delta_ because of its increased and H11-H4’s decreased interaction network with the RBD surface in the Delta variant.

Similar to H11-H4, H11-D4 was neither able to dislocate ACE2 from RBD_Delta_ nor considerably affect its interaction with RBD_Delta_. However, H11-D4 remained bound to RBD_Delta_ in the presence of PD, probably because it forms salt bridges R103-E35, R103-D38, and D108-K31 with ACE2 (Figure S5), while H11-H4 has a serine at position 103 and is unable to form these salt bridges. Collectively, these results suggest substantially reduced nanobody effectiveness against the Delta variant.

### Force-Induced Detachment of the Nanobodies from RBD

**Figure 5:**
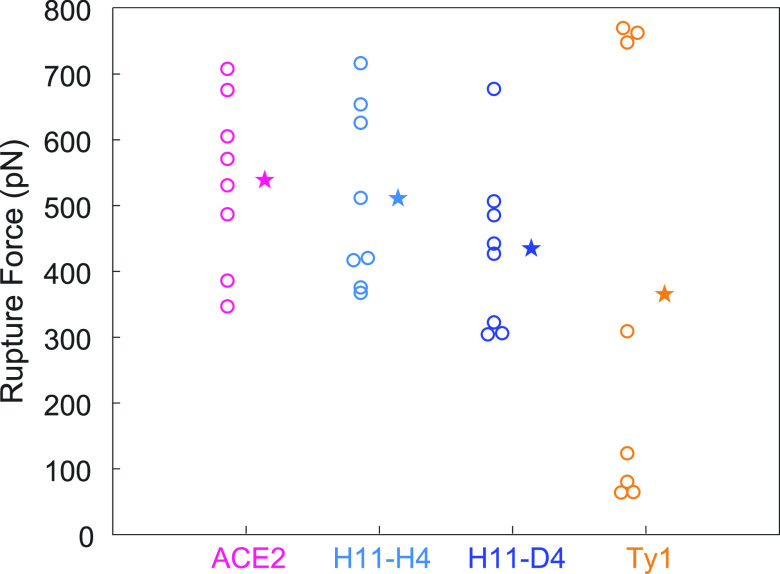
Rupture forces recorded from *in silico* pulling experiments. Each recorded rupture force is provided with a circle while their averages are shown with stars.

To estimate the binding strength of ACE2 and nanobodies to RBD_Delta_, we performed SMD simulations at loading rates (a spring constant of 10 pN/Å and a pulling velocity of 0.1 Å/ns) comparable to those used in high-speed AFM experiments.^34^ SMD simulations were performed by pulling the nanobodies at constant velocity along a vector pointing away from the binding interface (Figure S1). To be in accord with SMD studies on WT, Alpha, and Beta variants,^29^ RBD_Delta_ was pulled away from ACE2 to estimate ACE2’s binding strength to RBD_Delta_. Eight SMD simulations were performed for each system and their rupture forces were recorded (Figures 5 and S6). The average rupture forces were reduced by 5, 19, and 32% for H11-H4, H11-D4, and Ty1, respectively, when compared to ACE2. In comparison, we previously reported that H11-H4 has a higher binding strength for RBD_WT_, while H11-D4 and Ty1 have a slightly lower binding strength than that of ACE2. Collectively, our *in silico* pulling experiments indicate that nanobodies are not able to bind stronger to the RBD_Delta_ compared to ACE2, and they also suggest that especially for H11-H4 there is a strong tendency for the binding strengths to decrease relative to ACE2.

## Conclusions

In this study, we performed an extensive set of cMD simulations to investigate whether Delta variant mutations on the RBD of S protein affect its interactions with ACE2 and determined whether nanobodies are able to disrupt RBD_Delta_–PD interactions. To estimate rupture forces of the nanobodies and ACE2 from RBD_Delta_, we also performed SMD simulations at loading rates (1 pN/ns) comparable to high-speed AFM studies, which are ∼4–5 orders of magnitude lower than generally applied loading rates in SMD simulation. Thus, our SMD simulations provide a unique set of rupture forces that are directly comparable to experiments. Our simulations revealed that the Delta variant mutations lead to an increase in S–ACE2 interactions while decreasing the number of nanobody–S protein interactions. As a result, nanobodies H11-H4, H11-D4, and Ty1 have lower rupture forces from RBD_Delta_. H11-H4 and H11-D4 remained stably bound to RBD_Delta_, but they were unable to abrogate ACE2 binding when bound side by side on RBD_Delta_. In comparison, Ty1 exhibited floppy binding in one of the two simulations. Collectively, our results show the importance of identifying nanobodies for neutralizing specific variants of SARS-CoV-2^29,49^ and highlight the requirement of designing novel nanobodies to effectively neutralize the Delta variant.

In our previous study,^49^ we had shown that CR1 acts as the main anchor for SARS-CoV-2 S protein binding to ACE2, which is mainly facilitated through 10 hydrophobic interactions. Close inspection of the rupture events under load also shows that RBD_WT_ performs a zipper-like detachment, with CR1 detaching at the last in 80% of the simulations. In the Delta variant, CR1 forms two extra hydrophobic interactions, whereas CR3 gains one salt bridge and two hydrophobic interactions. CR1 detaches the last in five out of eight *in silico* pulling simulations, whereas CR3 detaches either the last or at the same time with CR1 in other simulations. This difference may be attributed to an increase in the number of pairwise interactions in CR3. Therefore, it may be critical to target both CR1 and CR3 to effectively inhibit S–ACE2 interactions of the Delta variant.

This manuscript presents a robust *in silico* strategy to predict the effectiveness of nanobodies to inhibit variant SARS-CoV-2 S protein’s RBDs, which may guide the design of novel nanobodies that can target specific SARS-CoV-2 variants. Well-established and proven *in silico* techniques, cMD and SMD simulations, are used to explore the binding strength of the nanobodies to RBD, and whether nanobody binding to RBD displaces ACE2 from RBD. The rupture force of RBD_WT_ from ACE2 was measured by AFM^51^ at loading rates of 5–6 orders smaller than those applied in our SMD simulations. This study reported rupture forces ranging from 70 to 105 pN. Although we applied loading rates 5–6 orders of magnitude larger than these experiments, we reported rupture forces with the same order of magnitude. Future experimental studies are needed to investigate how Delta mutations affect the RBD binding strength of H11-H4, H11-D4, and Ty1 nanobodies.

## Supporting Information Available

SMD simulation principles; the list of cMD and SMD simulations; steered and fixed atoms in SMD simulations; observation frequencies of interactions between RBD_Delta_ and PD and nanobodies for the combined trajectories; observation frequencies of interactions between RBD_Delta_ and PD and nanobodies for each simulation; pulling directions in SMD simulations; electrostatic interactions of RBD_Delta_ with ACE2 PD and nanobodies; change of the total number of pairwise interactions between RBD_Delta_-Ty1 during simulations; RMSD of ACE2s to their starting coordinates in the presence or absence of the nanobodies; salt bridges between ACE2 and H11-D4; and rupture forces from SMD simulations (PDF)
Movie S1 shows the Ty1 leaving its original binding mode (MP4)
Movie S2 shows the electrostatic repulsion between ACE2 and H11-H4 upon H11-H4 docking and H11-H4 dislocation for RBD_Delta_ (MP4)

## Notes

All data and software are available upon request to the corresponding author or may be accessed through the NSF MolSSI and BioExcel COVID-19 Molecular Structure and Therapeutics site at https://covid.molssi.org/.

## Abbreviations

μs: microsecond
ACE2: angiotensin-converting enzyme 2
AFM: atomic force microscopy
atm: standard atmosphere
Cα: α carbon
cMD: conventional molecular dynamics
CR: contact region
fs: femtosecond
MD: molecular dynamics
NAMD: nanoscale molecular dynamics
ns: nanosecond
NTD: n terminal domain
PD: peptidase domain
ps: picosecond
RBD: receptor-binding domain
RMSD: Root-mean-square deviation
mRNA: messenger ribonucleic acid
S: spike
SARS-CoV: severe acute respiratory syndrome-coronavirus
SMD: steered molecular dynamics
VMD: visual molecular dynamics
WT: wild type
