# Supplementary material for: SARS-CoV-2 Delta Variant Decreases Nanobody Binding and ACE2 Blocking Effectivity
Source: J Chem Inf Model. 2022 May 9;62(10):2490–8. doi: 10.1021/acs.jcim.1c01523 (PMC9113008; doi:10.1021/acs.jcim.1c01523)
Supplement: Supplementary file 1 — ci1c01523_si_001.pdf [file ci1c01523_si_001.pdf]

Supporting Information

SARS-CoV-2 Delta Variant Decreases Nanobody Binding and ACE2 Blocking Effectivity

*Mert Golcuk[1], Aysima Hacisuleyman[2], Sema Zeynep Yilmaz[1], Elhan Taka[1], Ahmet Yildiz[3,4], Mert Gur[1]**

[1] Department of Mechanical Engineering, Istanbul Technical University (ITU), 34437, Istanbul, Turkey

[2] Institute of Bioengineering, Swiss Federal Institute of Technology (EPFL), 1015, Lausanne, Switzerland

[3] Physics Department, University of California, 94720, Berkeley, CA, USA

[4] Department of Molecular and Cellular Biology, University of California, 94720, Berkeley, CA, USA

* Corresponding Author; e-mail: gurme@itu.edu.tr

SMD Simulations

In the SMD simulations a dummy atom, which is pulled at constant velocity along the pulling direction, is attached to the center of mass of "steered" atoms by a virtual spring. The resulting force **F** applied to the steered atoms depends on the instantaneous (at time *t*) center of mass coordinates of steered atoms **R** as follows,

$$\mathbf{F} = -\nabla U \tag{1}$$

$$U = \frac{1}{2} k[\mathbf{v}t - (\mathbf{R} - \mathbf{R}_0) \cdot \mathbf{n}]^2 \tag{2}$$

where U is the guiding potential, k is the spring constant, $\mathbf{v}$ is the pulling velocity, t is time, $\mathbf{n}$ is the direction of pulling and $\mathbf{R}_0$ is the coordinates of the center of mass of steered atoms at time t_0 [46]. Work performed is evaluated by integrating **F** over displacement ξ along the pulling direction.

Table S1. The list of cMD and SMD simulations.

MD ID	System	Simulation Type	Simulation Duration (ns)
1a-b	RBD_{Delta}-ACE2	cMD	(a) 400, (b) 400
2a-b	RBD_{Delta}-H11-H4	cMD	(a) 400, (b) 400
3a-b	RBD_{Delta}-H11-D4	cMD	(a) 400, (b) 400
4a-b	RBD_{Delta}-Ty1	cMD	(a) 400, (b) 400
5a-b	RBD_{Delta}-ACE2-H11-H4	cMD	(a) 400, (b) 400, (c) 400
6a-b	RBD_{Delta}-ACE2-H11-D4	cMD	(a) 400, (b) 400, (c) 400
7a-b	RBD_{Delta}-ACE2	SMD	(a1) 700, (b1) 650, (c1) 750, (d1)500<br>(a2) 600, (b2) 450, (c2) 550, (d2) 450
8a-b	RBD_{Delta}-H11-H4	SMD	(a1) 450, (b1) 450, (c1) 414, (d1) 550<br>(a2) 700, (b2) 448, (c2) 650, (d2) 750
9a-b	RBD_{Delta}-H11-D4	SMD	(a1) 550, (b1) 350, (c1) 450, (d1) 500<br>(a2) 700, (b2) 350, (c2) 550, (d2) 350
10a-b	RBD_{Delta}-Ty1	SMD	(a1) 790, (b1) 450, (c1) 756, (d1) 800<br>(a2) 150, (b2) 150, (c2) 100, (d2) 200

Table S2. Steered and fixed atoms in SMD simulations. C_α atoms of the listed residues were either steered or fixed.

System	Steered atoms	Fixed atoms
RBD-ACE2	K417-I418, G446-F456, Y473-A475, and N487-Y505 of RBD	S19-S43, T78-P84, Q325-N330, G352- I358, and P389-R393 of ACE2
RBD-H11- H4	R27-A33, R52-Y59, K74-T78, and H100-Y116 of H11-H4	R346, K444-F456, T470-I472, and G482-G496 of RBD
RBD-H11- D4	S25-A33, R52-Y59, and E100-Y116 of H11-D4	R346, K444-F456, T470-I472, and G482-G496 of RBD
RBD-Ty1	Q3-L6, A25-M36, Q41, R52-I60, I100-G113 of Ty1	R346-A352, K444-L455, I468-I472, and C480-G496 of RBD

Table S3. Observation frequencies of interactions between RBD$_{Delta}$ and PD and nanobodies in both set of simulations combined

	RBD$_{Delta}$-ACE2			RBD$_{Delta}$-H11-H4			RBD$_{Delta}$-H11-D4			RBD$_{Delta}$-Ty1		
	Name	All %	±s.d.	Name	All %	±s.d.	Name	All %	±s.d.	Name	S1%	±s.d.
Salt Bridge	R403-E37	75	1.0	E484-R52	100	0.1	E484-R52	100	0.1			
	K417-D30	96	1.1				K444-E100	34	3.1			
	E484-K31	77	1.0									
Hydrogen Bond	N487-Y83	65	0.3	E484-S57	55	0.3	F490-S104	61	0.6	Y449-Q112	54	1.2
	A475-S19	16	2.8	Q493-Y104	59	0.5	E484-S57	46	0.5	S349-Q3	19	1.9
	Q493-E35	28	0.7	S494-V102	70	0.2	Q493-S104	46	0.5	G447-Q112	20	1.4
	T500-Y41	36	0.3	Y449-H100	48	1.1	S494-V102	32	0.3	R452-V4	15	0.5
	T500-D355	42	0.5	F490-Y104	22	0.7				R452-R110	17	1.0
	Y505-E37	18	1.4	Q493-Y101	18	0.9				E484-Y35	32	0.8
										E484-L102	19	1.2
										Q493-L102	20	0.9
										Q493-S107	32	0.5
Hydrophobic Interactions	L455-T27	92	0.4	Y449-Y101	100	0.2	F456-L105	88	1.3	Y351-V4	100	0.7
	F456-T27	100	0.2	Y449-W112	100	0.3	Y489-L105	100	0.6	Y449-V109	78	0.5
	Y473-T27	100	0.3	L455-Y104	100	0.3	F490-V102	100	0.3	L455-L102	100	0.8
	A475-T27	100	0.5	F456-Y14	100	0.4	L492-V102	100	0.5	L455-L104	100	0.7
	A475-Y83	74	0.6	Y489-Y104	100	0.4				F456-L102	100	0.5
	F486-F28	99	0.9	Y489-L105	98	1.2				F456-L104	97	1.0
	F486-L79	100	0.5	F490-A32	90	0.6				V483-P55	100	0.4
	F486-M82	100	0.4	F490-V102	100	0.3				Y489-L102	100	0.4
	F486-Y83	99	0.6	L492-V102	100	0.4				Y489-L104	71	1.4
	Y489-T27	100	0.3	A475-Y104	22	1.2				F490-V4	100	0.7
	Y489-F28	100	0.3	V483-A58	47	1.1				F490-F29	100	0.7
	Y489-L79	93	0.7							F490-V34	100	0.3
	Y489-Y83	99	0.5							F490-V109	100	0.4
	V503-T324	82	1.2							L492-V4	100	0.5
	Y505-F356	68	0.8							L492-V109	100	0.3
	V445-L45	47	1.1									
Electrostatic Interactions	N487-Q24	53	0.5	Q493-V102	54	0.5	Y449-N101	18	0.8	Y351-Q3	17	1.3
	G496-K353	71	1.0	L492-Y104	40	0.3	N450-S30	16	1.6	Y449-R110	11	0.4
	T500-R357	51	0.7	Q493-S103	23	0.6	R452-S30	26	1.4	N450-Q112	15	0.7
	Y449-Q42	16	1.3				L492-S104	35	0.6	S452-Q3	18	1.4
	Y453-H34	26	0.5				Q493-V102	17	0.6	T470-G28	25	0.7
	A475-Q24	25	0.7				S494-N101	23	0.7	G482-S33	19	0.7
	Q493-K31	48	1.0							Q493-S108	26	0.7
	Q498-Q42	37	1.3							S494-S107	31	0.8
	Q498-K353	17	1.1									

Table S4. Observation frequencies of interactions between RBD_{Delta} and PD and nanobodies in each set of MD simulations.

	RBD_{Delta}-ACE2			RBD_{Delta}-H11-H4			RBD_{Delta}-H11-D4			RBD_{Delta}-Ty1	
	Name	S1%	S2%	Name	S1%	S2%	Name	S1%	S2%	Name	S1%
Salt Bridge	R403-E37	87	62	E484-R52	100	100	E484-R52	100	100		
	K417-D30	92	100				K444-E100	62	5		
	E484-K31	73	81								
Hydrogen Bond	N487-Y83	66	64	E484-S57	51	59	F490-S104	49	73	Y449-Q112	54
	A475-S19	31	2	Q493-Y104	59	59	E484-S57	53	40	S349-Q3	19
	Q493-E35	34	22	S494-V102	58	82	Q493-S104	32	60	G447-Q112	20
	T500-Y41	47	25	Y449-H100	14	83	S494-V102	54	10	R452-V4	15
	T500-D355	33	51	F490-Y104	14	29				R452-R110	17
	Y505-E37	20	16	Q493-Y101	16	20				E484-Y35	32
										E484-L102	19
										Q493-L102	20
										Q493-S107	32
Hydrophobic Interactions	L455-T27	94	90	Y449-Y101	100	100	F456-L105	90	86	Y351-V4	100
	F456-T27	100	100	Y449-W112	100	100	Y489-L105	100	100	Y449-V109	78
	Y473-T27	100	100	L455-Y104	100	100	F490-V102	100	100	L455-L102	100
	A475-T27	100	100	F456-Y104	100	100	L492-V102	100	100	L455-L104	100
	A475-Y83	73	74	Y489-Y104	100	100				F456-L102	100
	F486-F28	98	99	Y489-L105	96	100				F456-L104	97
	F486-L79	100	100	F490-A32	92	87				V483-P55	100
	F486-M82	100	100	F490-V102	100	100				Y489-L102	100
	F486-Y83	99	100	L492-V102	100	100				Y489-L104	71
	Y489-T27	100	100	A475-Y104	10	34				F490-V4	100
	Y489-F28	100	100	V483-A58	44	49				F490-F29	100
	Y489-L79	94	92							F490-V34	100
	Y489-Y83	99	98							F490-V109	100
	V503-T324	82	81							L492-V4	100
	Y505-F356	81	55							L492-V109	100
	V445-L45	45	48								
Electrostatic Interactions	N487-Q24	57	49	Q493-V102	44	65	Y449-N101	0	37	Y351-Q3	17
	G496-K353	86	55	L492-Y104	42	38	N450-S30	20	12	Y449-R110	11
	T500-R357	45	57	Q493-S103	30	16	R452-S30	41	11	N450-Q112	15
	Y449-Q42	21	10				L492-S104	24	47	S452-Q3	18
	Y453-H34	30	22				Q493-V102	32	2	T470-G28	25
	A475-Q24	27	24				S494-N101	4	42	G482-S33	19
	Q493-K31	63	33							Q493-S108	26
	Q498-Q42	43	32							S494-S107	31
	Q498-K353	7	27								

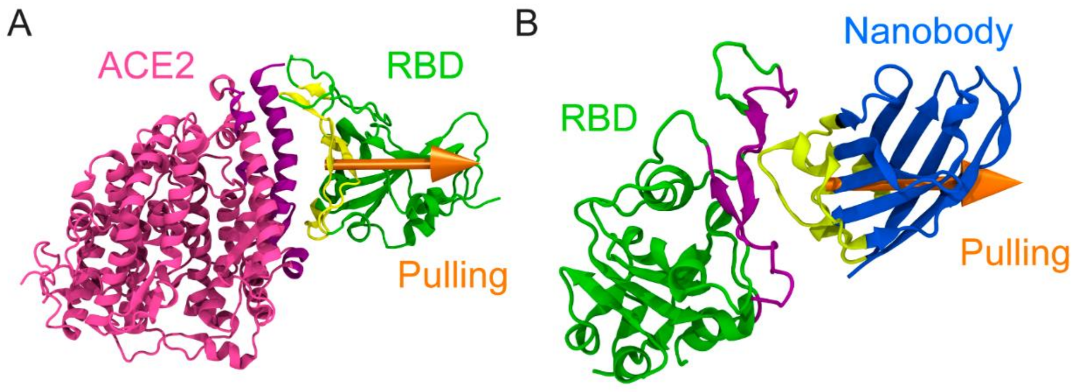

Figure S1. Pulling directions in SMD simulations. The pulling vectors pointing from the center of mass of fixed atoms (purple) to the center of mass of steered atoms (yellow) is shown for (A) RBD_{Delta}-PD and (B) RBD_{Delta} -nanobody simulations.

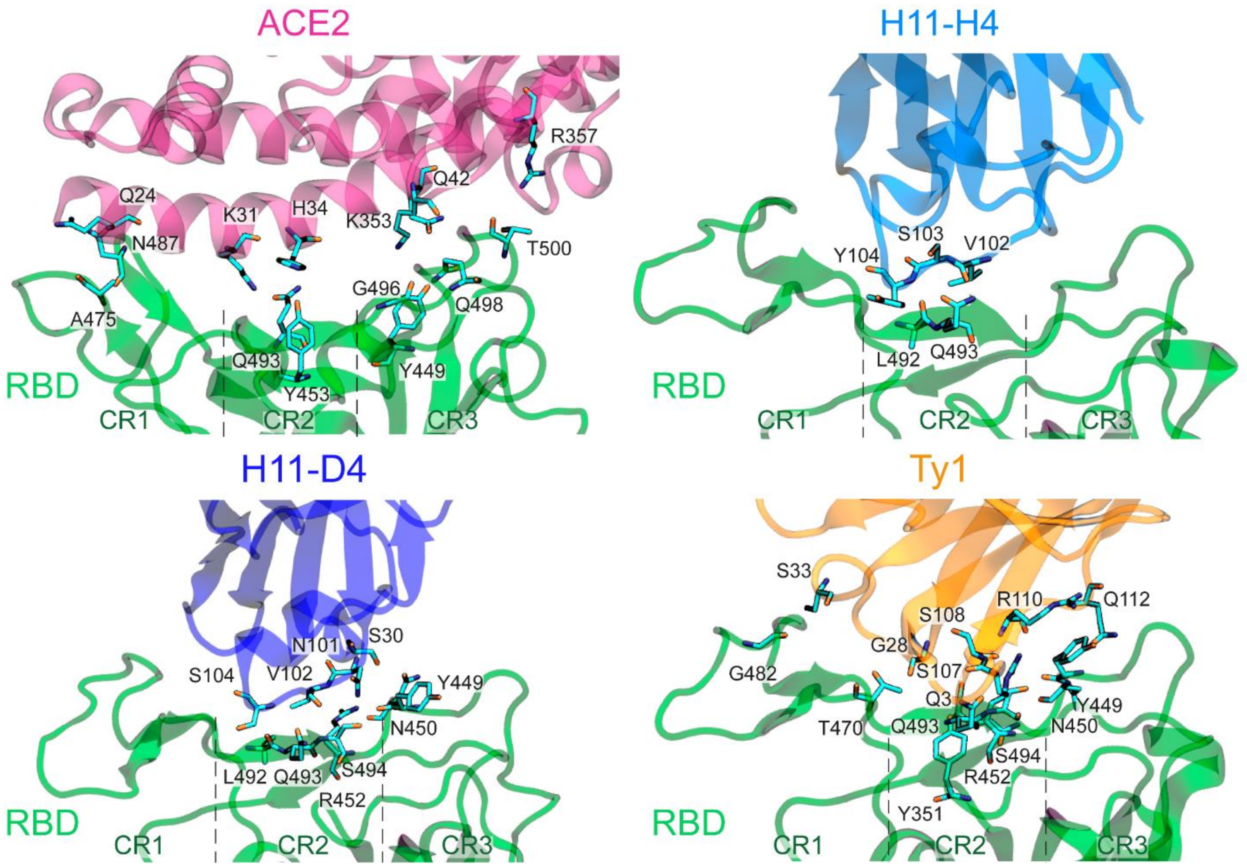

Figure S2. Electrostatic interactions of RBD_{Delta} with ACE2 PD and nanobodies.

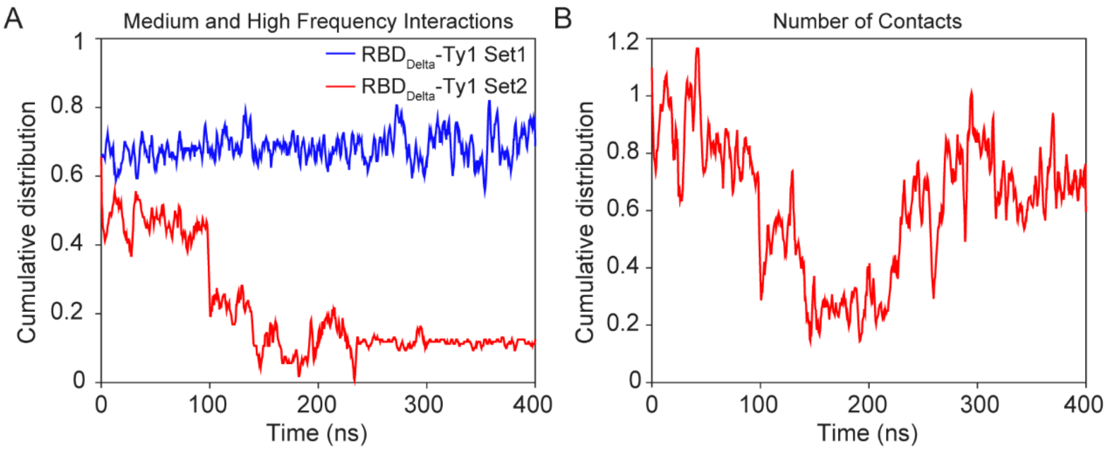

Figure S3. (A) Pairwise interactions between RBD_{Delta} and Ty1 were normalized by the total numbers of possible medium and high frequency interactions. While the original binding mode is conserved in the second set (MD 4b), Ty1 diverges from its starting binding pose within 100 ns and loses most of it initial interactions. (B) The changes in the number of contacts between RBD_{Delta} and Ty1 in the first set (MD 4a) of Ty1 simulations. For each conformation, all types of interaction (electrostatic interactions hydrogen bonds, hydrophobic interactions and salt bridges) were counted. Fluctuations in the number of contact sites indicate that various temporary binding modes are sampled during simulations.

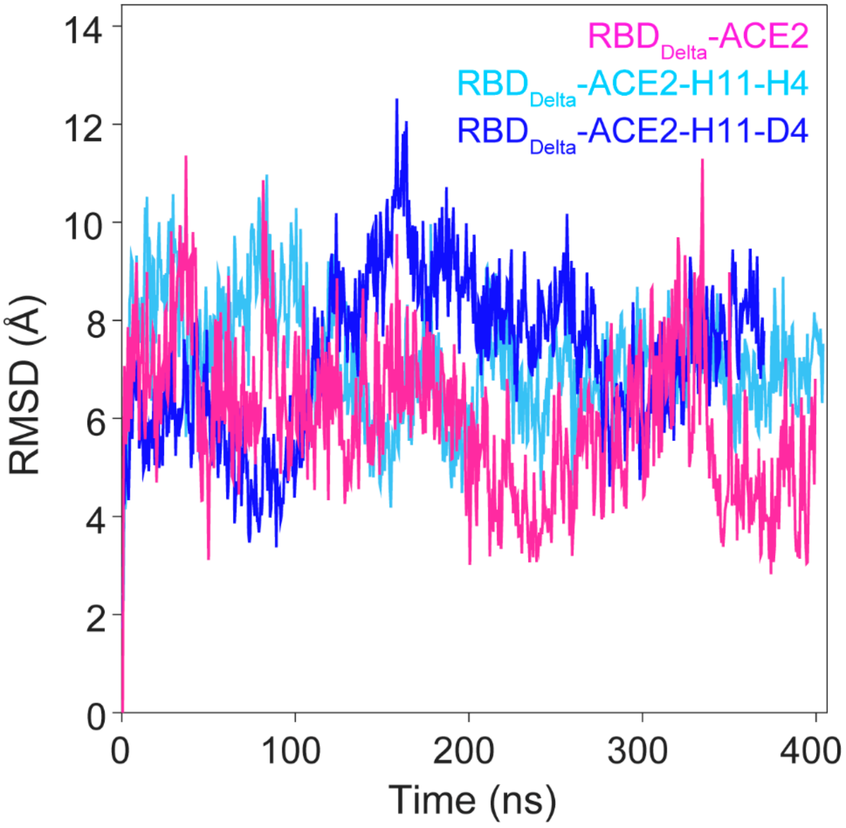

Figure S4. RMSD of ACE2s to their starting coordinates in the presence or absence of the nanobodies. All protein complexes were aligned via their RBD to their starting conformations, hence providing ACE2 fluctuations with respect to PD. Cα atoms were used for alignment and RMSD calculations.

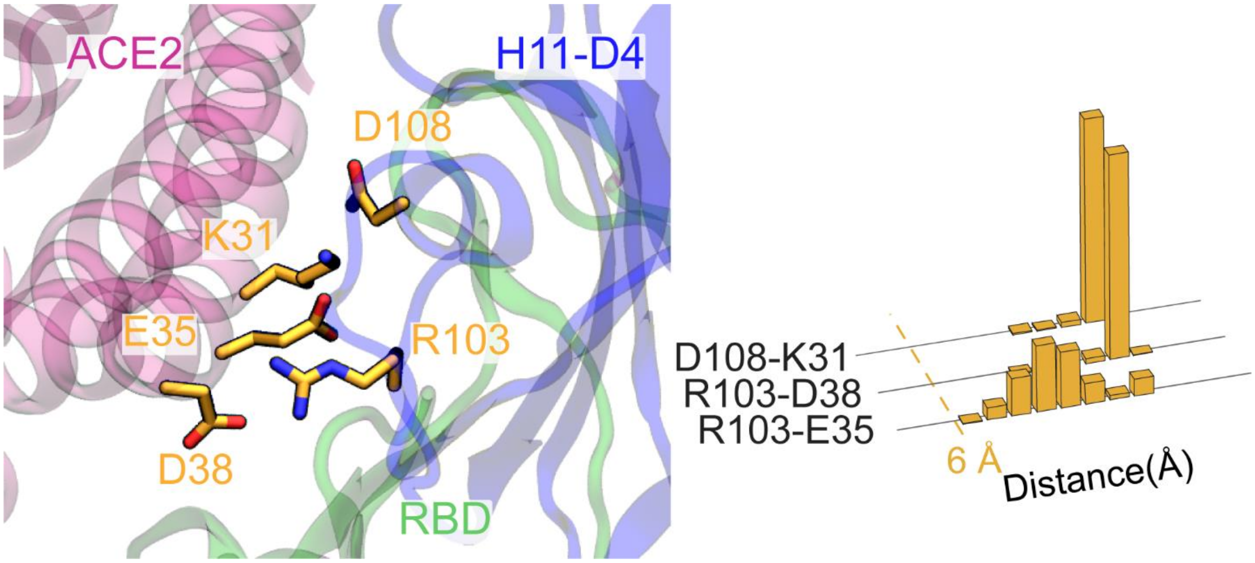

Figure S5. Salt bridges between ACE2 and H11-D4.

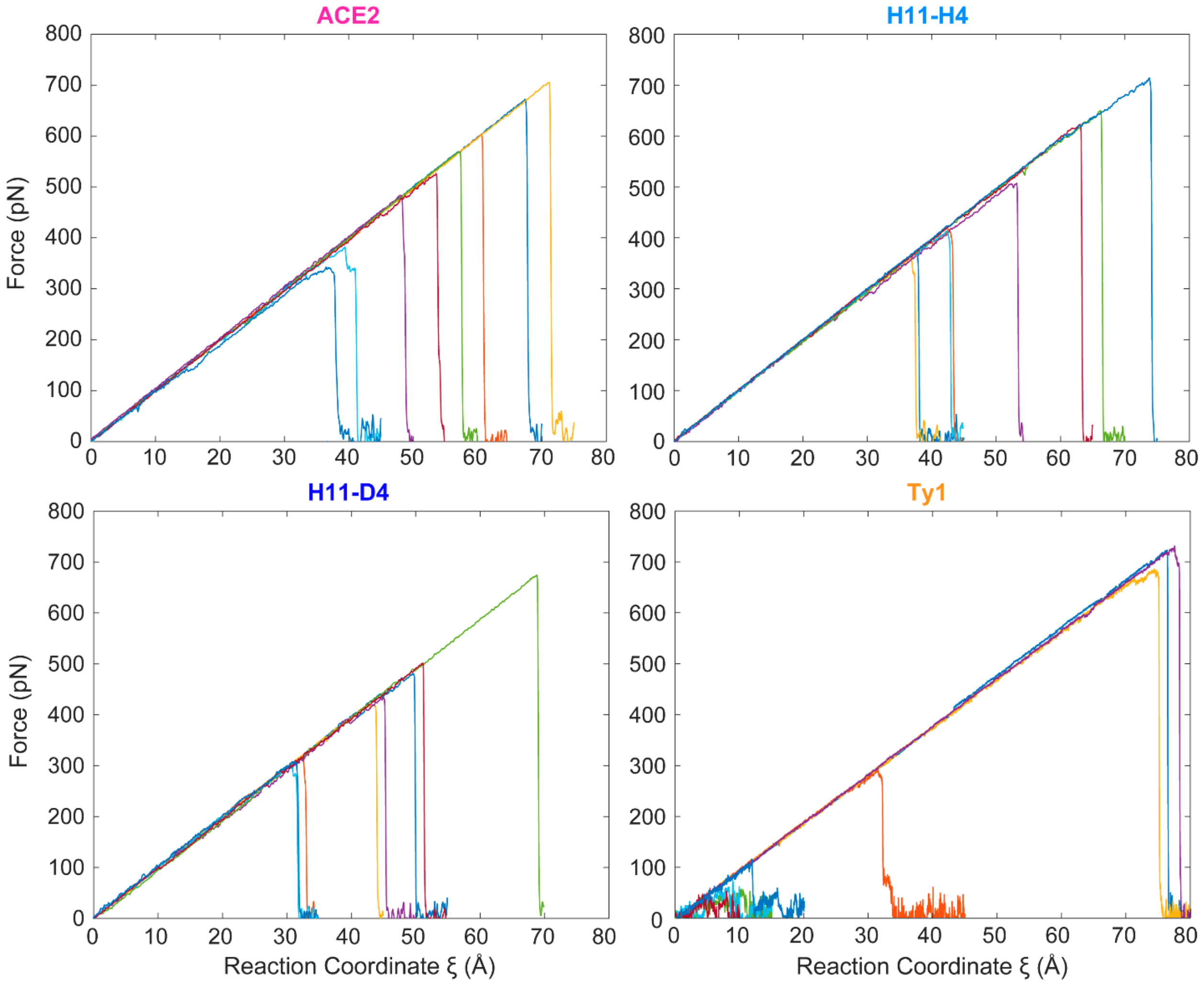

Figure S6. Rupture forces from SMD simulations where ACE2 and nanobodies are pulled from RBD_{Delta}. Rupture of ACE2 and nanobodies from RBD_{Delta} were obtained under linearly increasing forces. Rupture force is defined as the maximum recorded force before the force rapidly decreases to 0 pN.
